# Routine hypercapnic challenge after cervical spinal hemisection affects the size of phrenic motoneurons

**DOI:** 10.1038/s41598-023-40505-x

**Published:** 2023-08-25

**Authors:** Kenta Kawamura, Masaaki Kobayashi, Kazuhide Tomita

**Affiliations:** 1 Department of Physical Therapy, Ibaraki Prefectural University of Health Sciences, 4669-2 Ami, Ami-Machi, Inashiki-Gun, Ibaraki, 300-0394 Japan; 2 Graduate School of Health Science, Ibaraki Prefectural University of Health Sciences, 4669-2 Ami, Ami-Machi, Inashiki-Gun, Ibaraki, 300-0394 Japan

**Keywords:** Neuroscience, Anatomy, Neurology

## Abstract

After an individual experiences a cervical cord injury, the cell body's adaptation to the smaller size of phrenic motoneurons occurs within several weeks. It is not known whether a routine hypercapnic load can alter this adaptation of phrenic motoneurons. We investigated this question by using rats with high cervical cord hemisection. The rats were divided into four groups: control, hypercapnia, sham, and sham hypercapnia. Within 72 h post-hemisection, the hypercapnia groups began a hypercapnic challenge (20 min/day, 4 times/week for 3 weeks) with 7% CO_2_ under awake conditions. After the 3-week challenge, the phrenic motoneurons in all of the rats were retrogradely labeled with horseradish peroxidase, and the motoneuron sizes in each group were compared. The average diameter, cross-sectional area, and somal surface area of stained phrenic motoneurons as analyzed by software were significantly smaller in only the control group compared to the other groups. The histogram distribution was unimodal, with larger between-group size differences for motoneurons in the horizontal plane than in the transverse plane. Our findings indicate that a routine hypercapnic challenge may increase the input to phrenic motoneurons and alter the propensity for motoneuron adaptations.

## Introduction

After an individual experiences an injury to a high site at the cervical cord, motor paralysis of inspiratory muscles including the diaphragm and external intercostal muscles occurs, causing reduced lung capacity and a restriction of deep inspiration, which pose risks for the development of respiratory complications and atelectasis^1,2^. Respiratory muscle training is implemented in early-phase rehabilitation to help a patient with a cervical cord injury recover his or her respiratory function and prevent respiratory complications. Clinical studies have reported that respiratory muscle training after a cervical cord injury can increase a patient's respiratory muscle strength and vital capacity^3^.

Several studies of the neural recovery of respiratory function after cervical cord injury have used a model of higher C2 hemisection (C2HS). Since rats have phrenic motoneurons caudal to C3, the rat C2 hemisection model has been used and has been reported to show the recovery of diaphragm activity, phrenic nerve activity, and ventilation after C2HS^4–8^. The recovery of diaphragm and phrenic nerve activity and ventilatory volume has been shown to occur after C2HS^4–8^, and a cross-phrenic phenomenon (CPP) is considered a potential mechanism of this recovery^9,10^. The CPP is a latent pathway in the cervical cord that is gradually activated after a cervical cord injury. The presence of re-crossing inspiratory neurons has been confirmed in rats and cats^11,12^. Axonal sprouting and re-routing that may contribute to the neural recovery of respiratory function have also been described^13^.

Since the CPP pathway contributes to the recovery of respiratory function within a period of weeks to months, this pathway may contribute greatly to the recovery of respiratory function over the mid-to-long term^7^. The muscle activity of the rat diaphragm in the awake state was reported to be very slight on the first day of C2HS and to take 3 weeks to recover to the same level as that observed before the injury, suggesting that a temporary reduction in input from inspiratory neurons occurs during the first 1–2 weeks after this cervical cord injury^14^.

Phrenic motoneurons are also reported to show morphological alterations after C2HS, with a reduction in surface area and somal volume at 2 weeks post-C2HS^15^. The morphological alterations do not occur within 1 week after C2HS, indicating that these alterations occur gradually over time^16^. The mechanisms underlying these morphological alterations are not yet known, but one hypothesis is that morphological adaptations may occur as a result of the inactivity of the phrenic motoneurons^15,17^. Exposure to hypercapnic gas is known to activate the CPP^9^ and was reported to result in increased ventilation and phrenic nerve activity^4,7,18–20^. However, there is apparently no published investigation of whether a routine hypercapnic challenge can alter the adaptation of phrenic motoneurons.

We speculated that if motoneuron adaptations after a cervical cord injury are caused by a blockage of the input from inspiratory neurons to the phrenic motoneurons, then increasing the input to the phrenic motoneurons soon after a cervical cord injury may minimize the effects of such adaptations and aid in patients' recovery during the period before the adequate activation of the CPP pathway. We hypothesized that a daily routine hypercapnic challenge with high CO_2_ early after the injury in a C2HS model would increase the input to the phrenic motoneurons and inhibit motoneuron adaptation. We conducted the present study to (*i*) examine the possibility of preventing the post-cervical cord injury adaptations of phrenic motoneurons by implementing a hypercapnic challenge at an early timepoint after a C2HS in a rat model, and (*ii*) observe the morphological alterations of phrenic motoneurons in the model.

## Results

**Figure 1:**
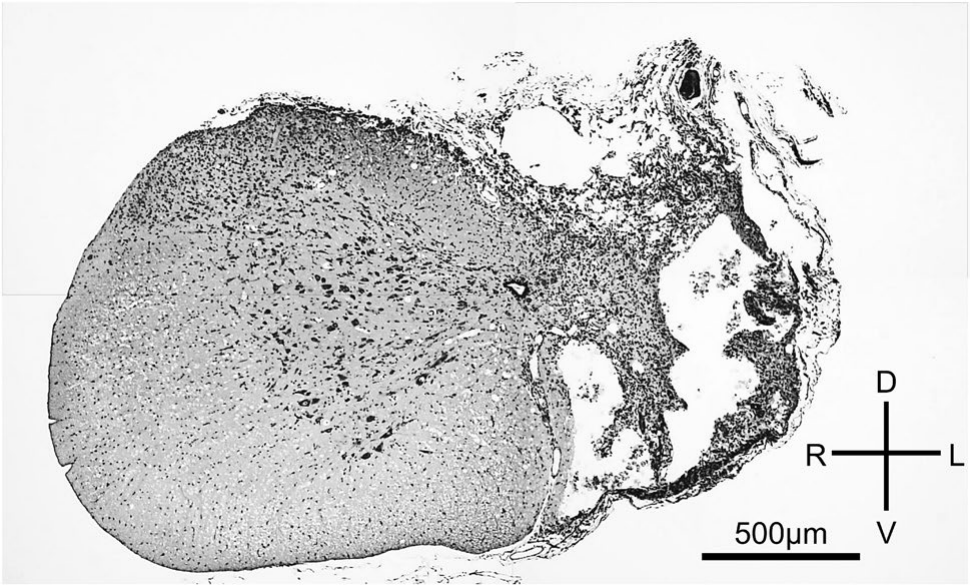
This section was hemisected at the left C2 level and stained with cresyl violet.

After the C2HS protocol, we were able to determine by visual observation and palpation that each rat's left diaphragm was paralyzed. The achievement of C2SH was confirmed by observation of a cross-section of the cervical spinal cord (Fig. 1). Whole-body plethysmography measurements on the third day after the C2HS revealed increases in respiratory parameters following the exposure to high CO_2_. The tidal volume increased from 1.1 ± 0.1 ml to 1.7 ± 0.3 ml (129.6% increase); the minute ventilation volume increased from 120.2 ± 21.9 ml/min to 283.9 ± 74.0 ml/min (177.9% increase), and the respiratory rate increased from 111.4 ± 16.1 breaths/min to 160.7 ± 15.3 breaths/min (138.8% increase).

### Phrenic motoneuron labeling

**Figure 2:**
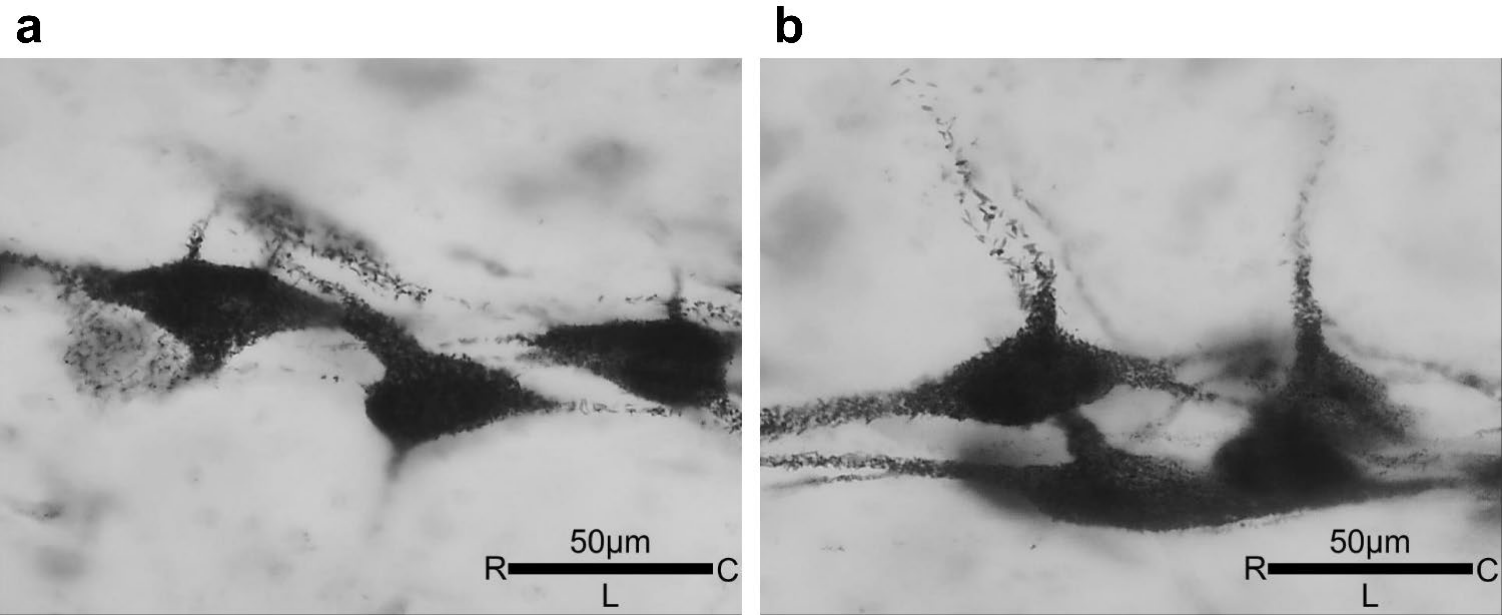
A typical image of horseradish peroxidase (HRP)-labeled phrenic motoneurons. The sections were in the horizontal plane. All motoneurons are ipsilateral to the cervical cord injury (left spinal cord). In each panel, the *left* is rostral, and the *bottom* is the lateral of spinal cord (**a**) control group. (**b**) hypercapnia group.

All phrenic motoneurons were distributed in the ventral horn of gray matter at the C3–C5/C6 cervical levels. The distribution of motoneurons was at more ventral-medial positions on the caudal side compared to the rostral side (C3–C4). Since it was difficult to obtain measurements of all of the neurons for the determination of the motoneurons' dense distribution, only the motoneurons for which the outer circumference could be measured were included in the analysis. Most of the the phrenic motoneurons were stained intensely, and thus their nuclei could not be identified; the axons of some of the motoneurons were identified (Fig. 2).

### Morphological differences in phrenic motoneurons in the transverse sections

The control group and hypercapnia group had mean ± standard error (SE) major axes at 29.3 ± 0.3 μm versus 32.3 ± 0.3 μm, minor axes at 19.6 ± 0.2 μm versus 23.1 ± 0.3 μm, average dia. at 24.5 ± 0.2 μm versus 26.1 ± 0.2 μm, and a cross-sectional area at 458.37 ± 7.0 μm^2^ versus 506.5 ± 6.7 μm^2^, respectively, demonstrating that all of these parameters were significantly larger in the hypercapnia group than the control group (all p < 0.001). The circularity values of the control and hypercapnia group were 0.82 ± 0.004 versus 0.79 ± 0.004, respectively, thus significantly more closely approximating a perfect circle in the control group (p < 0.001) (Table 1).

**Table 1:** Morphological features of the phrenic motoneurons in the control, hypercapnia, sham and sham hypercapnia group of rats.

		Control	Hypercapnia	Sham	Sham hypercapnia
Average dia., μm	T	24.5 ± 0.2	26.1 ± 0.2*		
	H	28.8 ± 0.2	30.9 ± 0.2*	30.7 ± 0.2*	30.2 ± 0.2*
Cross-section area, μm^2^	T	458.4 ± 7.0	506.5 ± 6.7*		
	H	589.5 ± 8.8	675.0 ± 6.2*	668.8 ± 9.0*	649.9 ± 8.4*
Somal surface area, μm^2^	H	8301.4 ± 133.6	9393.1 ± 86.6*	9309.1 ± 123.4*	9063.5 ± 118.6*
Major dia., μm	T	29.3 ± 0.3	32.3 ± 0.3*		
	H	37.5 ± 0.4	40.3 ± 0.3*	40.0 ± 0.5*	39.3 ± 0.4*
Minor dia., μm	T	19.6 ± 0.2	23.1 ± 0.3*		
	H	20.1 ± 0.2	21.5 ± 0.2*	21.4 ± 0.2*	21.1 ± 0.2*
Circularity ratio	T	0.82 ± 0.004	0.79 ± 0.004*		
	H	0.76 ± 0.005	0.75 ± 0.004	0.75 ± 0.006	0.73 ± 0.005*^,†^

Mean ± SE. Significant difference in multiple comparisons *vs. the control group, †sham vs. sham hypercapnia group. There were no significant differences between the hypercapnia and sham group or between the hypercapnia and sham hypercapnia group for all items. The numbers of phrenic motoneurons analyzed: control (n = 348), hypercapnia (n = 633), sham (n = 334), and sham hypercapnia (n = 386) group.

*H* horizontal sections, *T* transverse sections.

**Figure 3:**
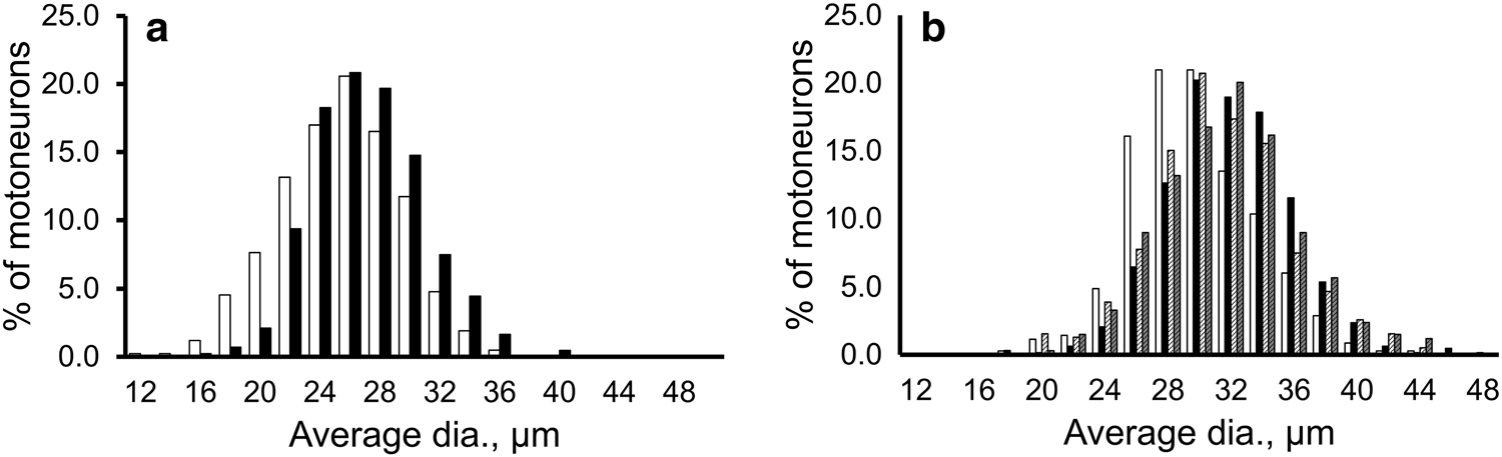
The histogram distribution of the average diameters of phrenic motoneurons. (**a**) Transverse sections. (**b**) Horizontal sections. *White bars:* control group. *Black:* hypercapnia group. *White striped*: sham group. *Gray striped*: sham hypercapnia group. The distribution of average dias. was larger in the hypercapnia, sham, and sham hypercapnia group than in the control group, and this was more visible in the horizontal sections.

**Figure 4:**
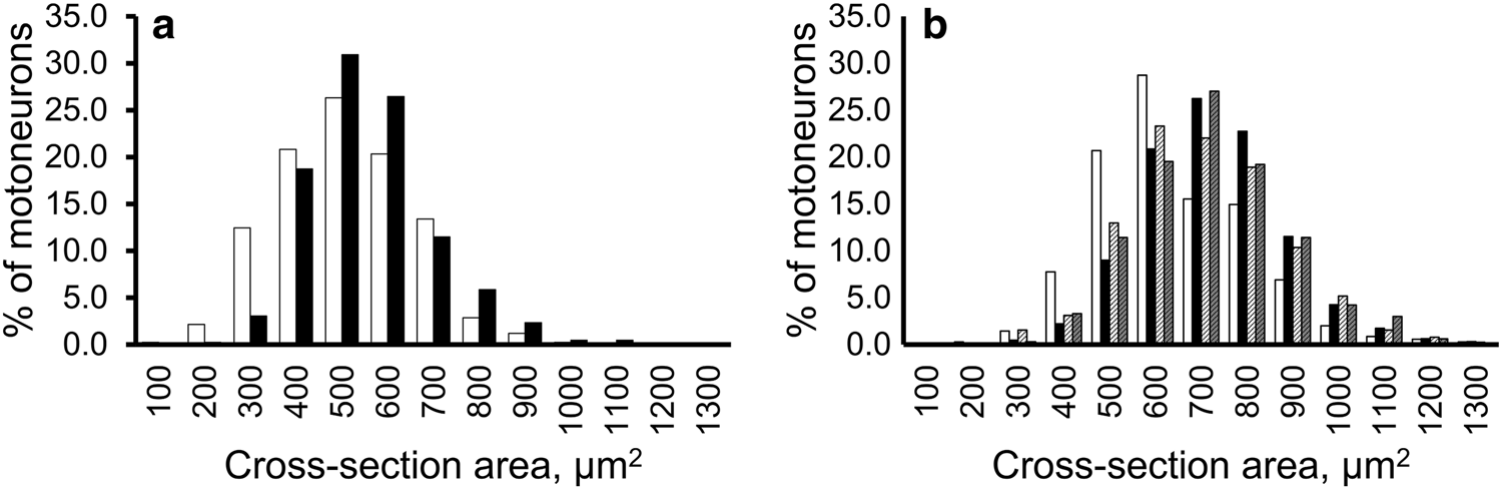
The histogram distribution of the cross-sectional areas of phrenic motoneurons. (**a**) Transverse sections. (**b**) Horizontal sections. *White bars:* control group. *Black:* hypercapnia group. *White striped*: sham group. *Gray striped*: sham hypercapnia group. The distribution of cross-sectional areas was larger in the hypercapnia, sham, and sham hypercapnia group than in the control group, and this was more visible in the horizontal sections.

**Figure 5:**
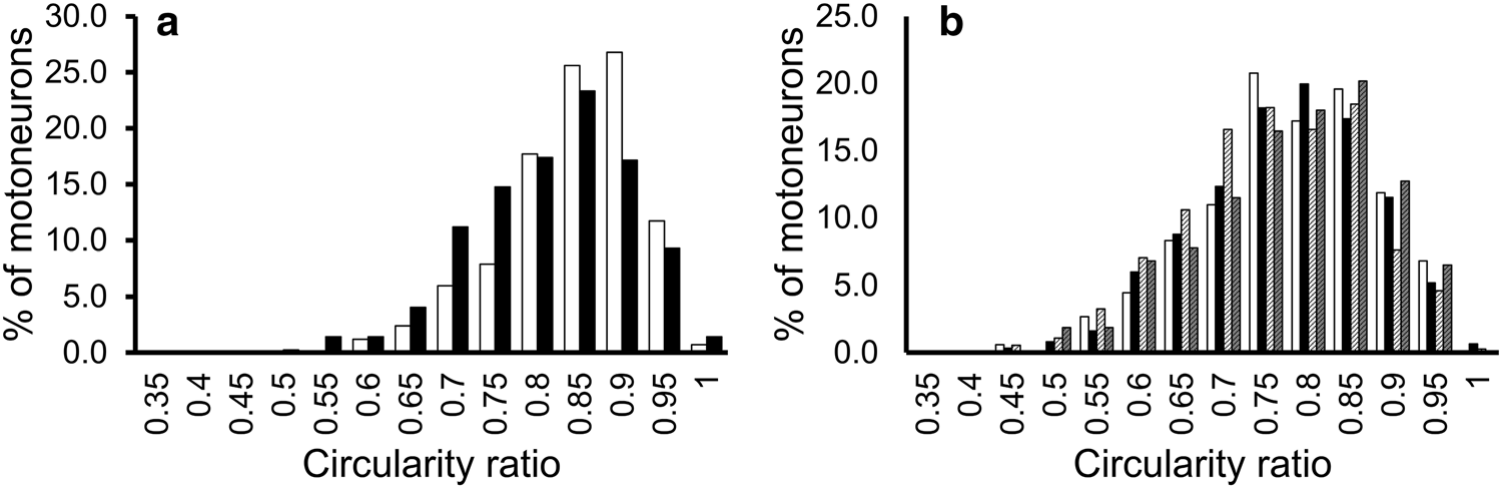
The histogram distribution of circularity ratio values. (**a**) Transverse sections. (**b**) Horizontal sections. *White bars:* control group. *Black:* hypercapnia group. *White striped*: sham group. *Gray striped*: sham hypercapnia group.

As illustrated in Figs. 3 and 4, the histograms of the distributions of average dias. and the cross-sectional areas showed that the peaks were similar in the hypercapnia and control group, but the hypercapnia group was biased slightly to the right (the larger side) and the control group was biased slightly to the left (the smaller side). In the histogram of circularity, the peaks and shapes in the two groups showed a similar trend (Fig. 5a). In the comparison of the circularity histograms of the cut sections, the transverse sections demonstrated higher circularity and a sharper histogram, and the horizontal sections demonstrated lower circularity and a gentler distribution compared to the transverse values.

### Morphological differences in phrenic motoneurons in the horizontal sections

The control, hypercapnia, sham, and sham hypercapnia group had major axes at 37.5 ± 0.4 μm, 40.3 ± 0.3 μm, 40.0 ± 0.5 μm, and 39.3 ± 0.4 μm, minor axes at 20.1 ± 0.2 μm, 21.5 ± 0.2 μm, 21.4 ± 0.2 μm and 21.1 ± 0.2 μm, average dia. at 28.8 ± 0.2 μm, 30.9 ± 0.2 μm, 30.7 ± 0.2 μm and 30.2 ± 0.2 μm, cross-sectional area at 589.5 ± 8.8 μm^2^, 675.0 ± 6.2 μm^2^, 668.8 ± 9.0 μm^2^ and 649.9 ± 8.4 μm^2^, and somal surface area at 8301.4 ± 133.6 μm^2^, 9393.1 ± 86.6 μm^2^, 9309.1 ± 123.4 μm^2^ and 9063.5 ± 118.6 μm^2^, respectively, demonstrating that the values of these size parameters were all significantly larger in the hypercapnia group compared to the control group (all p < 0.001) (Table 1). There were no significant differences between the hypercapnia and sham groups or between the hypercapnia and sham hypercapnia groups in any of the items. A comparison of the sham and sham hypercapnia groups revealed that the circularity ratio was significantly larger in the sham groups (p = 0.024). The circularity ratio of the sham hypercapnia group was significantly lower than those of the control and sham groups (p = 0.011, 0.025, respectively) (Table 1).

**Figure 6:**
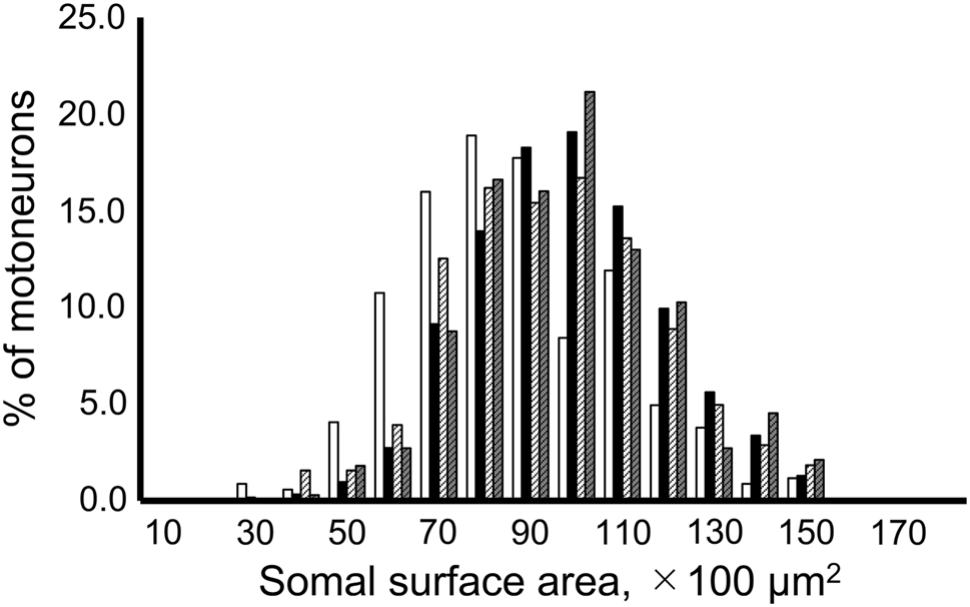
The histogram distribution of somal surface areas. Since phrenic motoneurons have a long elliptical shape in the rostro-caudal direction, we used the data from the horizontal sections to estimate the surface area. *White bars:* control group. *Black:* hypercapnia group. *White striped*: sham group. *Gray striped*: sham hypercapnia group. The distribution of somal surface areas was larger in the hypercapnia, sham, and sham hypercapnia group than in the control group.

The histograms for size showed similar peaks in the hypercapnia, sham, and sham hypercapnia groups, but the peak was to the left (the smaller side) in the control group (Figs. 3b, 4b, 6). The control and sham hypercapnia groups showed slightly higher values (0.7–0.75), but the histograms were generally similar (Fig. 5b).

## Discussion

We examined the morphological differences in phrenic motoneurons between control rats that underwent a C2HS, rats that underwent both a C2HS and a 3-week hypercapnic challenge, rats that underwent only a sham operation, and rats that underwent both the sham operation and the 3-week hypercapnic challenge. The C2HS rats exposed to high CO_2_ (i.e., the hypercapnic challenge) showed increased ventilation parameters. High CO_2_ may have increased the rats' descending inspiratory excitatory drive, since high CO_2_ was reported to increase phrenic nerve activity in addition to ventilation parameters^4,7,18–20^. All of the motoneuron size data were significantly smaller in the control group only and did not differ significantly among the other groups. These results demonstrate that a routine hypercapnic challenge after C2HS may affect the adaptations of phrenic motoneurons.

Unlike skeletal muscle, the diaphragm has few muscle spindles, and alpha motoneurons occupy most of the phrenic motoneurons; as in other studies^15,21^, our present histogram data showed unimodality. The sizes of the post-C2HS phrenic motoneurons in the horizontal sections were larger and the shape was more elliptical than those in the transverse sections. Compared to the horizontal sections, the cells' shape in the transverse sections was closer to a true circle. In this study, the sizes of the phrenic motoneurons in the transverse sections did not differ significantly from those in normal animals described in other studies^21,22^. The reported motor neuron major-axis lengths in horizontal sections from both normal animals and C2HS models tend to be larger than those in the present study^15,16,23^, although this may be due to differences in the measurement methods. An analysis by laser confocal microscopy could provide more accurate measurements of motoneuron sizes^24^, but since the purpose of our present investigation was to compare intervention effects, we believe that this is not a major problem in the interpretation of the present results.

C2HS models have been reported to have decreased phrenic motoneuron sizes compared to intact animals^15^, and our present findings support this concept. In addition to motoneuron size, it was reported that microstructural alterations of double synapses and increased phrenic dendrodendritic membrane appositions occur at 4 h after a cervical cord injury^9^. These alterations of phrenic motoneurons may be attributable to reduced motoneuron activity due to reduced input from the bulbospinal respiratory neuron. A 2020 study reported that a loss of excitatory glutamatergic innervation occurs in both small and large motoneurons after a C2HS, with a greater reduction in the innervation of small motoneurons^16^. Those results are also consistent with functional results showing no significant decrease in diaphragmatic electromyogram (EMG) activity during occlusion or sneezing, which recruits large motoneurons despite reduced ventilation volume and diaphragmatic EMG activity of resting breathing after cervical cord hemisection^7,25,26^. Based on the size principle, smaller motoneurons with slow conduction velocities are recruited earlier^27,28^, and this size principle pattern has been confirmed in the diaphragm motor units^29^. Whether this pattern follows the size principle after a C2HS is unclear, but it may compensate for the large decrease in synaptic input to the smaller motoneurons in order to facilitate recruitment with less input by reducing the size of the larger motoneurons. Despite the shift to smaller motoneurons, an EMG profile in which large motoneurons are recruited is maintained, suggesting that there is some reserve in the shift of motoneurons or that the amount of the synaptic input to motoneurons has a stronger influence on the EMG than the size.

However, we also observed that the sizes of the phrenic motoneurons in only the control group were smaller in both the horizontal and transverse sections. These results demonstrate that the increase of the input to the phrenic motoneurons by the hypercapnic challenge prevented the decrease in the size of the motoneurons. The CPP probably plays a major role in the medium- to long-term recovery of neural mechanisms. Investigations using anesthetized animals revealed that although ipsilateral phrenic-nerve firing occurs within weeks to months^7,30^, the amount of input does not recover over periods > 3 months^31^. In awake rats, the average amplitude of diaphragmatic EMG was observed to recover to 59.78% at 1 week and 92.38% at 2 weeks compared to pre-injury^14^.

Considering these studies, it appears that a period of several weeks to several months is required to return to pre-injury synaptic input levels. Since a hypercapnic challenge after a C2HS increases the action potential of the ipsilateral phrenic nerve^4,7,18–20^, we speculate that a hypercapnic challenge after a C2HS also increases descending inputs to phrenic motoneurons ipsilateral to the spinal hemisection. In the present study, the hypercapnia challenge was begun during the period of reduced synaptic input after the C2HS, which may have suppressed the neural adaptation of phrenic motoneurons. In general, the maintenance of the larger size of the phrenic motoneurons without a change to smaller sizes after a C2HS may be functionally disadvantageous because these motoneurons have decreased excitability. However, if the hypercapnic challenge induced sufficient plastic changes to allow sufficient recruitment without small changes in the motoneuron size, this could explain the present results.

Studies of the corticospinal tract in spinal cord injury models have reported that training induces plastic changes such as axon collateral sprouting^32^ and redistribution^33^ and enhanced synaptic connections of interneurons^34^. The phrenic motoneurons may not have needed to adapt to a smaller size because the hypercapnic challenge recovered inputs from the axonal side branches and interneurons at a relatively early phase. Another interesting observation in the present study is that the morphology of motoneurons was altered even though we used a limited number of hypercapnic sessions over a limited amount of time that increased excitatory input to motoneurons. The effect of limited numbers and amounts of the hypercapnic intervention could be applied in areas such as rehabilitation.

Several study limitations should be addressed. We analyzed only morphological characteristics and did not measure functional aspects. Functional as well as morphological alterations can be expected to occur after a C2HS, but the present investigation was not designed to examine such alterations. A methodological limitation of the study is that we were not able to observe all HRP-labeled motoneurons. Some motoneurons in the sections were excluded due to overlap with other motoneurons or because they had only part of the cell body. However, we believe that the effect of this exclusion is small based on the observed trends in the histogram distribution. In addition, since we examined only the size of the phrenic motoneurons, the mechanism underlying the motoneurons' adaptations resulting from the routine hypercapnic challenge after cervical cord injury are not clear from the present results. Data concerning the adaptation of dendrites and changes in the size of contralateral motoneurons could also be valuable. Finally, the mixed gases used for inducing hypercapnia in this study were intended to induce the activation of the CPP pathway and to increase the input to phrenic motoneurons; moreover, the oxygen concentration was set at a high value in order to simplify the facilitation of the complex pathways of hypoxia and hypercapnia. However, the long-term depression of respiratory activity may have limited the effectiveness of the intervention, and the hypercapnia could have been more effective depending on the method of loading^35,36^.

In conclusion, the rats that underwent an early post-C2HS hypercapnic challenge for 3 weeks did not exhibit smaller phrenic motoneuron sizes compared to the rats that did not undergo this intervention. It is probable that the hypercapnic challenge facilitated plastic alterations that resulted in the difference in motoneuron size.

## Methods

### Animals

Seventeen male Wistar rats (10–12 weeks old, weight 280–370 g; Japan SLC, Hamamatsu, Japan) were studied. The rats were housed individually cages in a controlled environment with a 12-h light/dark cycle (lights on at 07:00 h) with food and water ad libitum. All experimental procedures were performed according to the standards of the Guide for the Care and Use of Laboratory Animals published by the U.S. Department of Health and Human Services and the U.S. National Institutes of Health. The experimental protocols were approved by the Animal Care and Use Committee of Ibaraki Prefectural University of Health Sciences (approval no. 2021-2). This study is reported in accord with the ARRIVE guidelines.

### Spinal hemisection

The rats were divided into four groups: control (n = 6), hypercapnia (n = 10), sham (n = 3), and sham hypercapnia (n = 2). The control and hypercapnia group underwent a cervical cord hemisection procedure, i.e., a C2HS that was performed aseptically. For the C2HS, the rat was anesthetized with sevoflurane (initial dose 5.0%), and anesthesia was maintained with 3.0%–4.0% sevoflurane. The skin was incised from the first cervical vertebra to the seventh cervical vertebra, exposing the spine. The second cervical vertebra was laminectomized, and the left half of the cervical cord was hemisected from the midline with a micro-scalpel. The muscle and skin were sutured with 4–0 nylon sutures to close the wound. After surgery, penicillin G potassium was administered intramuscularly and saline was injected subcutaneously to help keep the rat warm on a heating pad. Aspirin (120 mg/kg) was administered orally in a timely manner according to the perceived postoperative pain status. The completeness of the cervical cord hemisection was confirmed by microscopy. The sham and sham hypercapnia group underwent the same procedure with the following exception: only a laminectomy of the vertebra was performed, and the cervical cord was not hemisected.

### Hypercapnia protocol

The hypercapnic challenge was started within 72 h after the C2HS procedure when the rat had recovered sufficiently. The challenge was conducted 4 times/week for 20 min each time. For the challenge, the hypercapnia group rat was placed in a chamber filled with high carbon dioxide (7% CO_2_, 50% O_2_, N_2_ balance). The 3-week hypercapnia challenge was performed. The control and sham group did not undergo any hypercapnia.

To confirm whether the exposure to high CO_2_ after the C2HS enhanced the rats' ventilation parameters, we used whole body plethysmography (Emka Technologies, Paris) on the 3rd postoperative day to measure the tidal volume, minute ventilation volume, and respiratory rate. Before the measurements began, the rat was left at rest for ≥ 20 min in the plethysmography chamber. Recording was done first for 20 min with the plethysmography chamber filled with room air, and then for 20 min with high CO_2_ (7% CO_2_, 50% O_2_, N_2_ balance). The data recorded during the last 5 min of each period were used for the analysis. Iox software (Emka Technologies) was used to calculate the degree of changes in the tidal volume, minute ventilation volume, and respiratory rate, with the resting state as 100%.

### Horseradish peroxidase labeling

After the end of the 3-week period, the rats in all groups were anesthetized with sevoflurane, and anesthesia was maintained with 3.0%–4.0% sevoflurane. The left neck was incised, the clavicle was retracted from the sternum, and the phrenic nerve was exposed. The phrenic nerve and the brachial nerve were carefully separated, and the separated phrenic nerve was cut as distal as possible. The phrenic nerve stump was inserted into a rubber micro-bag and left immersed in 30% horseradish peroxidase (HRP; PEO-131, Toyobo, Osaka, Japan) labeling solution for 2 h, with a change of the solution every hour. The surgical area, including the nerve stump, was then thoroughly washed, and the dissection was wrapped with an absorbent gelatin sponge. The muscle and skin of the surgical area were carefully sutured with 4–0 sutures. Postoperatively, the animals were kept warm and managed with analgesia as described for the spinal hemisection procedure.

The rats were allowed to awaken from the anesthesia, and 2 days later they underwent perfusion as follows. Under anesthesia with 5% sevoflurane, the rats were perfused transcardially with 200 mL of Ringer's solution followed by 300 mL of 1.25% glutaraldehyde and 1.0% paraformaldehyde in 0.1 M phosphate buffer (pH 7.4). The spinal cord was removed, and the spinal levels were identified. The spinal cord was immersed in fixative solution for ≥ 1 day and then in 30% sucrose buffer for 48 h.

Seven of the spinal cords were sectioned in the transverse plane (50-μm-thick sections) (control-group spinal cords, n = 3; hypercapnia-group spinal cords, n = 4), and the other 14 spinal cords were sectioned in the horizontal plane (100-μm-thick sections; control-group spinal cords, n = 3; hypercapnia-group spinal cords, n = 6; sham-group spinal cords, n = 3; sham hypercapnia-group spinal cords, n = 2). The sections were incubated with 3,3',5,5'- tetramethyl benzidine (T2885; Sigma-Aldrich, St. Louis, MO, USA) for the identification of neurons that endocytosed the HRP. Serial sections of each spinal cord were stained with neutral red eurhodin dye by the Nissel technique.

### Analysis of phrenic motoneurons

Microscopic images of the HRP-labeled phrenic motoneurons were taken, and each motoneuron was assigned a serial number. The sizes of the motoneurons were measured in the horizontal plane by Image J analysis software (U.S. National Institutes of Health, Bethesda, MD)^37^. We excluded the motoneurons in which the center of the cell body could not be observed and those in which the cell body overlapped and the morphology was difficult to observe. The motoneuron sizes were measured with the use of the major and minor axes of an ellipse fitted to the perimeter of the cell body (with the diameter measured perpendicular to the point halfway between the major and minor diameters); the average diameter (half of the sum of the major and minor diameters) was measured^38,39^. Circularity was calculated as follows: 4π × cross-sectional area/(perimeter)^2^. Circularity is a ratio with values that range from 0 to 1.0, with a value closer to 1.0 indicating a perfect circle and a value closer to 0 indicating an elongated shape. The cross-sectional area was measured from the values of the major and minor axes. We estimated the soma surface areas of phrenic motoneurons for a spheroid by using the lengths of the major and minor axes. No correction for tissue shrinkage was made. All data are expressed as the mean ± standard error (SE), and the data of each group were analyzed by an unpaired t-test or one-way ANOVA. Multiple comparisons adjusted by Bonferroni's method were performed only when there was a significant difference in the one-way ANOVA. All morphological data were analyzed with the use of SPSS ver. 28.0 software (SPSS; IBM, NY). The significance level was set at p < 0.05.

## Data Availability

The datasets analyzed during this study are available from the corresponding author on reasonable request.
